# Information and communications technology, health, and gender equality: Empirical evidence from a panel of Pacific developing economies

**DOI:** 10.1371/journal.pone.0269251

**Published:** 2022-06-15

**Authors:** Keshmeer Makun, Rup Singh, Sumeet Lal, Ronal Chand

**Affiliations:** 1 School of Accounting, Finance, and Economics, University of the South Pacific, Suva, Fiji; 2 School of Economics, Hiroshima University, Hiroshima, Japan; Universitat de Barcelona, SPAIN

## Abstract

Information and communications technology (ICT) has been widely embraced in many developing economies in recent times. Extant research reveals that ICT increases economic growth. Beyond economic growth, improved access to information, markets and economic opportunities via information and communications technology have the potential to influence other dimensions of public welfare. This study quantitatively examines the effects of ICT on selected health and gender dimensions of Pacific Island developing countries’ populations. The results show a statistically significant and positive impact of ICT on health and gender outcomes. Our results are robust with an alternative modeling approach, different control variables, and different measures of health and gender outcomes. We further establish that the health outcome of technology has a valid pass-through of income. The study suggests policy implications for the Pacific and other developing countries striving to enhance the health and gender outcomes of SGDs.

## 1. Introduction

Since the beginning of the new millennium, information and communications technology (especially mobile phones) has been increasingly embraced in developing and emerging market economies. Mobile technology has substantially helped people access information, in particular in rural and marginalized sections of the community that were not connected to any agile communication line previously. Mobile technology has also helped reduce transaction costs, leading to improvement and efficiency in market outcomes [1–5]. At the beginning of 2021, about 4.88 billion or about 62 percent of the world’s population were using mobile phones globally. About two-thirds of these mobile users are in developing countries. With a cellular subscription rate of over 100 on average, Asia and the Pacific region has one of the highest mobile penetration rates [6].

In the Pacific, mobile technology is not only a communication device but is also a primary conduit of economic participation and means of accessing welfare-enhancing goods and services. This is especially relevant to remote areas where about half of the population lives and the provision of public services is limited. ICT is already playing a significant role in tackling many structural and geographical challenges in the island nations. A number of key factors are pushing this change. In particular, the deregulation of telecommunications sector since 2013. ICT uptake is also accelerated by regions’ youth bulge as about one-fifth of the population in the Pacific is aged 15–24. The recent social and economic challenges brought about by the COVID19 pandemic show a huge uptake of mobile technology, which is not only used to access public services including health and gender related services but also used in the policy response to reduce infection, for example, contact tracing. ICT has enabled responses by facilitating large-scale involvement and mass collaboration across the region and national borders [7]. For example, incorporating new organizations in an existing humanitarian collaboration network (e.g., volunteer and technical groups that aid during crises, such as pandemics and natural disasters); enabling innovative ways of providing surveillance and transmitting timely information and assistance (e.g., digital money wallet, online self-support groups, online therapy for COVID-19 infected patients) and also facilitating diverse public engagement to counter the spread of misinformation [8]. On the other hand, the crisis also highlights the limitations in the use of ICT due to a lack of innovations, skills, training, and infrastructure [9]. The distributional nature of the population, small markets, lack of human resources, and cost of connectivity also hamper the dissemination of ICT in Pacific Island countries.

A growing number of studies have analyzed the impacts of mobile technology on productivity, market access, prices, economic growth, agriculture production, and income [10–16]. (; However, ICT can potentially affect various other aspects of public welfare like health [17, 18] and gender empowerment [8, 19, 20]. [21] argue that on-hand access to various computer sites within the community or in schools is one of the effective ways to close the gap in accessibility and usage of ICT, which does not only aid in enhancing the status of the girls and women, but also boosts societal empowerment, equality, and development. [22–24] examine the use of information and communication technology, including mobile use for boosting health efficiency and patient care. Such wider implications of ICT are crucial, particularly given the mission to achieve Sustainable Development Goals (SDGs). Specifically, SGD 3 and 5, good health and wellbeing, and gender equality, respectively, are arguably equally important if not more than the conventional economic indicators.

A number of research and agency reports discuss how ICT could impact food security, health outcomes, gender, and other aspects of welfare [5, 19, 25–27]. However, the quantitative analysis on this subject remains void. There is also a view that developing economies should focus on the basic health care system, education, and income support for all rather than technology infrastructure [28]. This disagreement existed perhaps due to under-performing health systems and high gender disparity in many poor and less developed countries. Concerns about ICT use are important when it begins to have an adverse effect on society and the beneficial effect does not lead to long-term social and economic improvement. This is essential when long-run outcomes depend on other factors like education and training, affordability, and access, which is becoming an important element in the adoption of technology.

In this paper, we attempt to disentangle the socio-economic implications of ICT and contribute to the literature from the perspective of developing Pacific Island economies. Specifically, we empirically analyze the relationship between ICT and health outcomes and ICT and gender equality in a panel data setting. Like other developing economies, mobile technology is being adopted quite rapidly in small Pacific Island developing countries in the last few decades and is commonly used even among rural and poor households. Besides its valuable effect on the commerce and economy, the advent of ICT is also envisaged to have impacted socio-economic characteristics. However, there is no quantitative evidence to support appropriate policy interventions. To the extent that we know, this paper is the first to examine ICT’s implication on health and gender outcomes for Pacific Island Countries (PICs). We use the virtual Random-effect panel and robust least square (MM-based) methods in this study to examine the relationship between ICT and socio-economic (health and gender) outcomes. Further, it is important to note that identifying a clear relationship between mobile technology and socio-economic outcomes is hard in the self-selection situation. As a result, we also examine possible mechanisms through which ICTs impact health outcomes in PICs. Given the dearth of quantitative analysis on the social implications of ICT, we believe that this study will make an essential contribution to the literature and potentially stimulate future similar ICT-related research.

The remainder of the paper is organized as follows. The following section provides a literature review of ICT, health, and gender outcomes. Section 3 provides data and indicators used in the quantitative analysis. Section 4 outlines the econometric methodology for the study. Section 5 presents the results, and the last section 6 provides the conclusion with policy implications.

## 2. Literature review

In this section, we provide a literature review on the socio-economic (health and gender) impact of information and communication technology. First, we provide a brief theoretical overview of the relationship between ICT, health, and gender. Secondly, we review the extant literature on ICT and health outcomes. We examine the literature both, from a positive perspective as well as the differing views and findings from interdisciplinary literature. Third, we provide the literature on the nexus between ICT and gender outcomes.

It has been argued that technology has the potential to improve health and gender outcomes [18, 29]. Conceptually, the health implications of ICT or mobile technology can take place through different channels. For example, better economic opportunities facilitated by ICT and the associated rise in income are typically linked to improvements in social conditions and quality of life [30] ICT also provides access to geographically challenged communities and support for health care workers in reaching out to them to provide relevant services. Access to a wide variety of services through digital technology can improve public health education and advice on dealing with and mitigating the consequence of risk factors, which leads to improved health outcomes [31–33] Globally, digital technologies are being used to aid public health response. A recent example is the ongoing COVID-19 pandemic where technology is playing a fundamental role in areas like population monitoring, case identification, contact tracing, and intervention assessment based on morbidity data and public consultation [34]. To the extent of surviving in the current COVID19 business worl [35], show possible business development possibilities through the use of ICT. Further, gender roles in society can also drive health outcomes [21]. For those countries where gender gaps in health are shaped by individual and societal norms that tend to undervalue women’s health, relevant interventions which include ICT platforms for effective, innovative, and faster dissemination of messages are needed to reinforce the importance of women and girls for the reduction in health gaps [36]. As females tend to be more concerned about well-being in terms of food and spending on health care relative to males, gender empowerment can improve health outcomes even if there are no income gains [33] Hence, empowering females will generally drive innovation for inclusive structural change, which would fast-track movement toward the SDGs [37].

Focusing on various case scenarios [38, 39], provide a review of the link between digital technology and the health system and the patient-doctor relationship. The review indicates that digital technology has the capacity to enhance health care systems and personal health outcomes by engendering friendly partnerships. Similarly [24], in a theoretical preposition suggests that ICT can be used to improve health information systems. Computer-based diagnostics and ICT applications can improve the performance of the health care system. Similar findings are reported in [40–42].

Further, the evolution of online health platforms and e-health contributes to improvement in population health. The web-based essential health information boosts the knowledge of people and enables them to take control of their health [42] Not only that, e-Health has the ability to advance care and offer new services for people with multimorbidity while allowing policymakers to organize and incorporate different elements of better care. For instance, expand clinician decision-making and the quality of care through support systems and categories of risk to identify the most composite cases and allow for more pre-emptive responses [43]. Digital technology can also improve communication, procurement of medicine, and monitoring of patients. [44] piloting a drug supply chain show that ICTs have huge potential to bring efficiency in stock management in the health system. Tsai et al. (2017) show that e-learning facilitated by ICT in addition to face-to-face interaction enhances health education in Taiwan.

[45] provide an overview of mobile text platforms as a communication tool in behavioral change for disease management and control. The authors show evidence of managing some chronic diseases like diabetes control, reduction in smoking, and weight loss. They suggest that text massaging can be a vital tool to reduce health burden by providing timely curative and preventive support. “m-health” in their words, offers interactive communication and a range of opportunities to improve health-related outcomes including in remote areas and capitalize on young population behavior given their ability to access and use of mobile technology. Similarly, [46] focusing on developing countries provide a review report on the use of SMS-supported technology for disease surveillance and management. Generally, they find evidence that mobile SMS applications are an accepted mechanism among the population for disease control interventions.

Besides positive effects on health, there are also some criticisms of the applications of ICT. Conservative societies question the nature and quality of the online information available on various platforms that can be misleading and misinterpreted, leading to adverse health outcomes. It is argued that wrong information can produce anxiety and even lead to serious illness and ultimate mortality [47]. There are some socioeconomic groups that do not have full or reliable and proper access to the information. In this situation, it can further exacerbate health disparities among socioeconomic groups. [38] show that online misinformation can lead to unnecessary anxiety and visits to hospitals costing time and money. While [48] find a positive correlation between personal mobile and patient’s healthcare decisions, they found that health-related phone use delays access to public health experts. [32] show that uneven technology diffusion increases health care inequalities. The inequalities disproportionately discriminate against poor and non-adopters of technology than affluent households [49]. Similarly [18], suggest that while mobile phones have the potential to be a vital therapeutic resource and are already used by people to secure health care, they can be impacted by limited access to technology, lack of ‘digital capital’ and poor health service provisions.

According to [37],the digital divide is the gap between those who have and those that do not have access to computers and the internet. Digital divide is also classified under four successive types of access, which are: (i) motivational, (ii) physical, (iii) skills, and (iv) usage. The fact is that digital inequalities persist and vary by sector (community and individual levels) and elites continue to govern the content of various ICTs even though this power has been altered in certain ways by digital media through asymmetries between elites and people [50]. As such [51], contends that technology and its different aspects should be understood and explained in the context of a specific situation because technology is not a socially neutral artifact. [52] explains that while the exponential diffusion of mobile phones in Africa has chartered new ways for people to interface with power through the lenses of the ‘liberation technology’ agenda, it has neglected numerous actors and networks that intervene in shaping governance processes. [53] explain that wealth and connectivity are necessary but not sufficient factor for explaining inequalities in geographies of user-generated information that are accessible to some people and places. He suggests a combination of the total amount of human sites, activities, processes, practices of interest, the nature of the broader information ecosystem, and the societal attitudes toward learning and the sharing of information as contributing factors.

Studies have also discussed the potential impact of ICT on gender roles [20, 29, 54, 55]. Improved market access through mobile technology enhances information availability, which could enable women to make independent decisions [31]. Conceptually, women are mostly constrained in access to markets and information [56]. Giving access to ICT can possibly benefit women by allowing them to equally participate in mainstream economic activity. It is also suggested that to harness ICT for effective development, ICT should be mainstreamed as a broader mechanism for enhancing opportunity and empowering the poor [56]. Explicitly identifying and addressing the gender dimension of ICT such as access, use, capacity building opportunities, and employment can serve as a strong mechanism for cultural, political, and social liberation of women and the advancement of gender equality [10, 20, 57, 58].

Interactions between phone usage, female empowerment, and poverty is complex [59]. It is found that while there are changes in everyday routines, in the context of female empowerment they find little support for mobile as a tool for transformative improvement in Africa. Similarly, [20, 47] argue that while the phone eases communication and improves social ties, it is difficult to circumvent the classified nature of society where ‘class and place of residence are typical social makers in the process of social networking. [55] also provide an alternative perspective, where communication using ICT generates social conflicts, hence enhancing the understanding of the broader impact of technology- not necessarily from the development perspective. Various gender issues including women’s political participation, employment opportunities, education, and violence against women have been highlighted in Pacific Island countries. These issues have been one of the most challenging issues in the social, economic, and cultural development of PICs. While some progress has been made, for instance, in education, this has not translated into improved opportunities and jobs for women. The labor market survey shows that while women’s participation in the market is increasing [60], it is lower than that of their male counterparts [19].

Finally, it is obvious from the literature that the social implications of ICT on health and gender outcomes are attracting the interest of researchers and policymakers in recent times. While the extant literature is not homogenous and provides some information and comfort, the real impact and measure of ICT on health and gender aspect is not that apparent. A quantitative analysis of the relationship between ICT and health and gender dimension is imperative to provide greater insight into this phenomenon. The available evidence of health and gender implications of ICT is scarce and focuses on specific countries, particularly large and developed countries, where it is hard to generalize the findings. This paper, therefore, quantitatively analyses this relationship in the context of Pacific Island developing countries, which is an unexplored setting in the literature.

## 3. Data, variables, and measurement

The quantitative analysis is based on unbalanced panel data set. Based on the availability of data, the study includes five Pacific Island countries namely, Fiji, Samoa, Solomon Islands, Tonga, and Vanuatu. Lack of consistent data on key social indicators is a common feature in small Pacific island countries. With limited resources, it is hard to produce various social statistics required for measuring progress. However, certain progress in some major countries reporting enabled us to obtain some consistent data. The study period covers 2000 to 2018. The key explanatory variable of focus is information and communications technology (ICT). We proxy ICT using mobile phone subscriptions per 100 inhabitants. The data is sourced from International Telecommunication Union (ITU) [6].

With respect to our outcome variables, we are interested in health outcomes and gender equality. Health outcome is measured in different ways [19, 27] including dietary-based indicators, access to healthcare facilities, and anthropometrical measures. Given the focus here is to examine how ICT influences health outcomes, anyone indicator may or may not be a good indicator. In this study, we use the life expectancy index and infant mortality rate as our health indicators. Life expectancy refers to the average years lived from the year of birth. The infant mortality rate is measured by death under one year of age per thousand live births. The data for these two variables is sourced from World Health Organization (WHO) and World Development Indicators (WDI).

Gender equality is measured by the female labor force participation rate and female employment. In assessing gender equality or the social situation of women, literature commonly uses individual measures on education, health, employment, and political and economic opportunities [39, 57, 60–62]. Given the absence of consistent composite indices, we focus on how ICT use may impact individual indicators like female labor force participation and female employment. A larger proportion of female labor and female employment can be an indication of a higher level of women empowerment [19]. The data on the female labor force and employment is obtained from World Bank’s WDI database.

### 3.1. Control variables

Besides ICT, we also included additional control variables in our health and gender regression models. For instance, human capital is expected to play a crucial part in enhancing health care outcomes and gender equality. An educated and qualified individual has a high chance of getting a better job and earning income. Further, human capital accumulation enables people to read and understand health-related information and prevent risk factors. Human capital also has a vital role in gender empowerment. For instance, increasing education not only benefits female health but also improves their ability to participate in other economic opportunities. In this paper, we have used health and gender indicators that are likely to be influenced by human capital. It is measured by secondary school enrollment and is sourced from Penn World Tables- an online database.

In health regression, we also include health expenditure. An important determinant of health outcomes is public health expenditure [63]. With adequate investment in healthcare facilities and primary healthcare services, the prevalence of diseases (communicable and non-communicable) can be avoided and result in better health outcomes. In this study, we use health expenditure as a percent of GDP, which is available on the World Bank’s WDI database 2020.

For gender equality, we also introduce tourism earnings as an additional control variable. Pacific Island countries heavy reliance on tourism for employment and income led to substantial social and economic change in the last couple of decades. Thus, we also use the tourism indicator (tourism earnings) from WDI to see if the gender dimension can be enhanced through tourism-related investment. Human capital is also included in gender regression.

Summary statistics of the variables are shown in Table 1 and correlation analysis in Table 2 for health and gender outcomes. Samoa has the largest mean life expectancy index followed by Tonga, Solomon Islands, Vanuatu, and Fiji. The average female labor force participation rate is highest in the Solomon Islands at about 64% and lowest in Samoa at 25%. The mean mobile phone subscription is highest in Fiji (63 per 100 inhabitants) followed by Tonga, Samoa, Vanuatu, and the Solomon Islands. The Jarque-Bera test shows if series are normally distributed. The correlation analysis shows that ICT is positively correlated with health at about 0.13 and with gender at about 0.15. There is also a consistent positive correlation of health and gender with other independent variables such as health expenditure and human capital.

**Table 1:** Summary statistics for Pacific Island countries.

**Fiji**							**Samoa**					
	**LE**	**LFPRF**	**MOB**	**HEX**	**HC**	**TE**	**LE**	**LFPRF**	**MOB**	**HEX**	**HC**	**TE**
Mean	0.716	40.676	63.851	3.248	2.572	772.000	0.789	25.961	36.879	5.113	81.784	106.000
Median	0.716	40.591	72.974	3.262	2.589	774.000	0.789	25.956	47.821	4.945	82.031	113.000
Maximum	0.727	46.471	119.749	3.536	2.681	1190.000	0.816	26.474	77.625	6.228	88.429	169.000
Minimum	0.703	36.258	6.789	2.905	2.422	291.000	0.755	25.444	1.425	3.933	74.416	39.000
Std. Dev.	0.007	3.418	41.832	0.169	0.082	272.000	0.021	0.356	26.369	0.675	4.640	44.282
Skewness	-0.115	0.216	-0.140	-0.496	-0.422	-0.394	-0.151	0.009	-0.151	0.115	0.008	-0.206
Kurtosis	2.058	1.660	1.458	2.616	1.921	2.006	1.565	1.631	1.533	2.191	1.675	1.563
Jar-Bera (p)	0.703	0.476	0.398	0.654	0.495	0.547	0.534	0.579	0.520	0.813	0.599	0.521
Observations	18	18	18	18	18	18	14	14	14	14	14	14
**Solomon Islands**							**Tonga**					
** **	**LE**	**LFPRF**	**MOB**	**HEX**	**HC**	**TE**	**LE**	**LFPRF**	**MOB**	**HEX**	**HC**	**TE**
Mean	0.772	64.304	26.709	7.269	25.624	38.168	0.770	47.500	43.181	4.427	103.389	24.894
Median	0.773	64.343	7.833	6.889	21.311	36.200	0.769	47.524	49.919	4.525	103.365	17.650
Maximum	0.810	65.368	73.161	10.780	48.544	79.000	0.780	48.142	105.824	6.132	119.319	52.700
Minimum	0.730	63.462	0.228	4.741	13.885	0.800	0.764	46.310	0.184	2.927	94.527	5.900
Std. Dev.	0.025	0.636	30.152	1.990	10.510	28.438	0.005	0.483	29.356	0.834	5.946	16.327
Skewness	-0.082	0.127	0.467	0.336	1.019	-0.081	0.628	-1.007	0.144	0.076	0.788	0.483
Kurtosis	1.798	1.630	1.399	1.757	3.003	1.451	2.077	3.725	2.449	2.404	4.057	1.653
Jar-Bera (p)	0.576	0.483	0.276	0.473	0.211	0.403	0.402	0.180	0.865	0.868	0.259	0.357
Observations	18	18	18	18	18	18	18	18	18	18	18	18
**Vanuatu**							**Panel summary statistics**					
** **	**LE**	**LFPRF**	**MOB**	**HEX**	**HC**	**TE**	**LE**	**LFPRF**	**MOB**	**HEX**	**HC**	**TE**
Mean	0.749	62.000	29.389	3.497	17.470	171.000	0.758	48.811	40.403	4.721	45.155	229.000
Median	0.750	62.122	13.934	3.425	12.729	165.000	0.765	47.551	47.821	4.167	27.972	72.900
Maximum	0.767	62.457	71.942	4.158	39.547	314.000	0.816	65.368	119.749	10.780	119.319	1.190
Minimum	0.729	61.358	0.185	3.049	6.687	58.000	0.703	25.444	0.184	2.905	2.422	0.800
Std. Dev.	0.012	0.290	28.148	0.342	11.016	88.976	0.029	13.818	34.063	1.807	40.505	318.000
Skewness	-0.149	-0.589	0.276	0.725	1.000	0.139	-0.032	-0.356	0.433	1.561	0.419	1.676
Kurtosis	1.856	2.569	1.248	2.644	2.439	1.453	2.212	1.843	2.182	5.092	1.489	4.467
Jar-Bera (p)	0.628	0.592	0.325	0.476	0.237	0.439	0.335	0.040	0.084	0.000	0.005	0.000
Observations	16	16	16	16	16	16	84	84	84	84	84	84

Note: LE is life expectancy index, MOB is the mobile phone, representing ICT, HEX is the health expenditure, HC is human capital, LFPRF is labor force participation rate for females, and TE is the tourism earnings.

**Table 2:** Correlation for health and gender outcome.

Panel correlation for health outcome						Panel correlation for gender outcome					
	LE	MOB	HEX	FI	HC		LFPRF	MOB	FDI	TE	HC
**LE**	1.000					**LFPRF**	1.000				
**MOB**	0.129	1.000				**MOB**	0.151	1.000			
**HEX**	0.387	-0.269	1.000			**FDI**	0.234	0.101	1.000		
**FI**	0.510	-0.100	-0.012	1.000		**TE**	0.232	0.595	0.369	1.000	
**HC**	0.665	0.018	0.087	0.405	1.000	**HC**	0.399	0.010	-0.458	-0.546	1.000

Note: LE is life expectancy index, MOB is the mobile phone, representing ICT, HEX is the health expenditure, FI is food import, HC is human capital, LFPRF is labor force participation rate for females, FDI is foreign direct investment, and TE is the tourism earnings.

## 4. Econometric model

The objective of this paper is to empirically examine the possible relationship of mobile ICT with health outcomes and gender equality. Thus, it is assumed that the health and gender dimensions are engendered with the following model:

$$Y_{ti}=\beta_{0}+\beta_{1}ICT_{ti}+\beta_{2}X_{ti}+\varepsilon_{ti}$$ (1)

Where *Y*_*ti*_ is the dependent or outcome variable referring to time *t* and country *i*. *ICT*_*ti*_ is the independent variable of attention. *X*_*ti*_ is the vector of additional contextual variables likely to influence health and gender outcomes and *ε*_*ti*_ is the random white noise (error) with a symmetric distribution.

Given we have two outcome variables (health and gender equality), we estimate separate regression for health and gender. *β*_1_ is elasticity estimate of the ICT, which is of particular interest. A positive and statistically significant estimate would indicate that mobile ICT is positively linked to health and gender equality. We further probe this relationship after considering other variables included in *X*_*ti*_.

### 4.1. Analyzing possible mechanism

Further, we analyze possible mechanisms for ICT and health outcomes. The effect of Mobile ICT on income and gender is fairly clear and straightforward. Information and communications technology reduces cost and improves access to information and markets, which increases efficiency, output, and income [11]. Similarly, mobile ICT empowers women and girls who are disproportionately constrained in terms of access to information and economic opportunities [58]. However, the health outcomes of mobile ICT are relatively less candid. The health of the population may change through the different processes including gender equality and income. Given that casual trails is hard to identify, thus we use the following regression models to obtain further intuition into possible channels of mobile ICTs implications:

$$Health_{ti}=\delta_{0}+\delta_{1}Inc_{ti}+\delta_{2}Gen_{ti}+\delta_{3}ICT_{ti}+X_{ti}+\varepsilon_{ti}$$ (2)

Where *Health*_*ti*_ refers to health indicator measured by the life expectancy index. *Inc*_*ti*_ is the income measured by GDP per capita, and *Gen*_*ti*_ is gender equality. *δ*_1_ and *δ*_2_ are coefficients of income and gender equality. It is expected that they are positive and significantly associated with health. *δ*_3_ is estimate for ICT and test if mobile phone positively impacts health while controlling for *Inc*_*ti*_ and *Gen*_*ti*_. Statistically insignificant *δ*_3_ would imply that we can cautiously say that Mobile ICTs’ effect on health is mainly passing through income and gender equality. However, positive and statistically significant *δ*_3_ would indicate other channels may also have a role. For example, mobile ICT could directly influence health. For instance, ICT can possibly enhance access to customize health information, which would influence healthy living through dietary and food choices.

### 4.2. Estimation methodology

We begin with the one-way fixed effect regression model (FEM) of Gujarati and Porter (2009. In a FEM model, each country, *i*, is allowed to have its own time-invariant (hence the name fixed effect) intercept (*β*_0*i*_ instead of *β*_0_) while assuming that the slope coefficients are constant across time. The FEM equation is presented as follows:

$$Y_{ti}=\beta_{0i}+\beta_{1}ICT_{ti}+\beta_{2}HEX_{ti}+\beta_{3}HC_{ti}+\beta_{4}TE_{ti}+\mu_{ti}$$ (3)

If we relax the assumption that *β*_0*i*_ is time-invariant and substitute it with a random variable with a mean value of *β*_0_ (no *i* subscript) such that:

$$\beta_{0i}=\beta_{o}+\varepsilon_{i}$$ (4)

Where *ε*_*i*_ is a random error with mean 0 and variance, $\sigma_{\tau }^{2}$. The resulting method is called the one-way random effect model (REM), also known as the error components model (ECM) because the composite error term, *w*_*ti*_, consists of two (or more) error components. The REM equation is presented as follows:

$$Y_{ti}=\beta_{0}+\beta_{1}ICT_{ti}+\beta_{2}HEX_{ti}+\beta_{3}HC_{ti}+\beta_{4}TE_{ti}+w_{ti}$$ (5)

Where *w*_*ti*_ = *ε*_*i*_+*μ*_*ti*_. The composite residual, *w*_*ti*_ has two elements: *ε*_*i*_, which is the cross-section or individual-specific error and *μ*_*ti*_, which is the collective time series and the cross-section error component.

The question of which model (FEM vs. REM) is preferable is based on the assumption one makes about the likely correlation between the individual, cross-section, error component, *ε*_*i*_, and the regressors. If it is assumed that *ε*_*i*_ and the regressors are uncorrelated, the random effect model would be appropriate, whereas if *ε*_*i*_ and the regressors are correlated, the fixed effect model can be appropriate. Before proceeding with the estimation, we also address some common concerns with respect to the series under study. We conducted stationarity tests on the PICs panel and ensured that the variables in the study are stationary. We apply the panel unit root test [64–66]. Collinearity is less likely in a panel since the country cross-section adds variability [67].

## 5. Results and discussion

We start our quantitative analysis by using panel fixed and random effect estimation. Although the elasticity coefficients are consistent in both estimations, we verify the appropriateness of the two models. In panel analysis, researchers routinely employ fixed and random estimation models and select the appropriate model based on [68] test. The test statistics generated by Hausman have an asymptotic *X*^2^ distribution. We carry out redundant fixed and correlated random effects Hausman test. The null hypothesis for the fixed effect is that it is redundant whereas the null hypothesis for the random effect is that omitted variables are uncorrelated. The fixed and random effect tests are shown in Table 3.

**Table 3:** Fixed and random effect test.

Panel A: Redundant fixed effect				
Tests	Statistics			Prob value
Period Fixed	13.136***			0.061
Period Chi-square	22.341***			0.043
Cross section fixed	1.391			0.183
Cross section Chi-square	0.826			0.563
Panel B: Correlated random effect				
Test summary		Chi-square stats	Prob value	
Period random		5.611	0.433	

Note: Null hypothesis for the fixed effect is that it is redundant. The null hypothesis for random effect is that omitted variables are uncorrelated with explanatory variables.

*** indicates a 10% significance level.

For the fixed effect test in Panel A, the significant probability values indicate that the null is rejected and that period effects are not redundant. However, cross-section effects are insignificant, implying that the effects are redundant. This means that omitted variable issue is not fixed for the panel of countries across time. On the other hand, the correlated random effect test [68] in Panel B shows that the random effect model is uncorrelated, indicating random effect model is appropriate. Thus, the result from the random effect is presented and discussed in the paper.

## 5.1. Health outcomes of ICT

Although the sample of countries is from the same geographical region and similar demographic and socio-economic parameters, the specific health and gender outcomes can differ between countries. To consider country differences, we also include country dummies in the random effect model. However, the result remains fairly the same and country dummies are found to be statistically insignificant. We also run these regressions with MM-based Robust Least Square estimation to compare the consistency of the estimates.

Table 4 reports the results of the relationship between ICT and health using random effect (RE) and Robust Least Square (RLS) estimator. We estimate three variants of Eq (1) to test the robustness of the result of ICT on the health outcomes for the panel of five Pacific Island countries. The results show that ICT (mobile phone use) is positively associated with life expectancy, which is an indicator of healthy living. In all specifications, mobile use is positive and significant. With the RE estimator and controlling for other factors, mobile use has a 0.003 percent higher life expectancy. The coefficient for mobile phone use in the RLS estimation is similar in magnitude change and statistically significant. Although this proposition is not been examined earlier for PICs [5, 27], hypothesized that technology can contribute to healthy living. Access to customized health care advice through mobile devices would enable the population to better look after themselves. Health expenditure as a percent of GDP and human capital is found to have a positive and statistically significant impact on life expectancy in all models.

**Table 4:** Results of ICT and health (life expectancy).

	Health (life expectancy)				Health (life expectancy)		
		RE estimation			RLS estimation		
		Model 1	Model 2	Model 3	Model 1	Model 2	Model 3
*Mobile phone use*		0.003 (0.004)*	0.003 (0.016)*	0.002 (0.088)***	0.002 (0.017)**	0.039 (0.000)*	0.045 (0.000)*
*Health exp*		-	0.017 (0.003)*	0.021 (0.000)	-	0.016 (0.000)*	0.202 (0.000)*
*Human capital*		-	-	0.019 (0.000)	-	-	0.021 (0.009)**
*Trend*		0.001 (0.000)*	0.001 (0.000)*	0.001 (0.000)*	0.007 (0.000)*	0.004 (0.001)*	0.005 (0.000)*
*Constant*		-0.133 (0.000)*	-0.122 (0.000)*	0.171 (0.000)*	-	-	-
*R-square*		0.588	0.676	0.686	0.54	0.582	0.592
*Observations*		84	84	84	84	84	84

Notes: The estimated coefficients are shown with probability values in brackets. RE is a Random effect. RLS is Robust Least Square.

* is p<0.01

** is p<0.05 and

*** is p<0.1.

The result of health regression with the infant mortality rate as a dependent variable is shown in Table 5. We use this specification to check the robustness of the relationship between ICT and health outcomes. As expected, mobile phone use is negatively associated with the infant mortality rate, implying the use of technology reduces the infant mortality rate. In all the specifications, mobile use is negative and statistically significant. Technologies like mobile phones and other personalized digital gadgets help deliver health-related information, monitor the health of mothers and infants, and enhance self-management of health. For instance, through the development of ICT tools, such as the MyKana app, which tracks the dietary intake on regular basis, people are able to monitor their calorie intake and develop improved health regimes. With the additional control variable, we find that in the final model (3) health expenditure as a percent of GDP has a significant positive relationship with the infant mortality rate. This is consistent with the government’s planned resource allocation for basic primary health care services such as ante-natal care, immunization, nutrition, and health programmes for communities. In comparison to the findings from the literature, [63], for example, show a positive but insignificant impact of health expenditure on the crude death rate in Pacific Island countries. Human capital is inversely related to the infant mortality rate in PICs. This is plausible as knowledge and information gained via technology about health, would make the population more cautious and practice healthy living [69]. This finding also suggests the need for training and education of the population with respect to ICT skills. The result for mobile use and health-related expenditure in RLS estimation is consistent and similar to RE estimates.

**Table 5:** Results of ICT and health (infant mortality rate).

	Health (infant mortality rate)				Health (infant mortality rate)		
		RE estimation			RLS estimation		
		Model 1	Model 2	Model 3	Model 1	Model 2	Model 3
*Mobile phone use*		-0.020 (0.014)**	-0.018 (0.006)**	- 0.072 (0.000)*	-0.028 (0.006)**	-0.049 (0.000)*	-0.293 (0.001)*
*Health exp*		-	0.017 (0.003)*	0.036 (0.082)***	-	0.016 (0.000)**	0.088 (0.000)*
*Human capital*		-	-	-0.103 (0.000)*	-	-	-0.107 (0.050)**
*Trend*		-0.001 (0.026)**	0.001 (0.615)	0.008 (0.001)**	-0.043 (0.000)*	-0.021 (0.043)**	0.023 (0.017)**
*Constant*		-1.303 (0.000)*	1.212 (0.000)*	-1.418 (0.000)*	-	-	-
*R-square*		0.57	0.68	0.664	0.484	0.592	0.675
*Observations*		84	84	84	84	84	84

Notes: The estimated coefficients are shown with probability values in brackets. RE is a Random effect. RLS is Robust Least Square.

* is p<0.01

** is p<0.05 and

*** is p<0.1.

### 5.2. Gender outcomes of ICT

Table 6 shows the results of ICT on gender equality. Although insignificant, initially (Model 1) the effect of mobile phone use is positively associated with the female labor force, which is an indicator of gender equality. In the subsequent models (2 and 3) we introduce additional economic control variables. We find that ICT and gender have a statistically significant and positive relationship. This finding potentially suggests that other economic fundamentals need to be in place for ICT to be effective in reducing gender gaps [56]. The coefficient estimate of mobile phone use is fairly consistent across the models and with a different estimator. Assuming everything else constant, mobile subscription increases the female labor force participation rate by 0.033 percent, which is about a 2 percent increase over the mean female labor force participation rate in PICs. This effect of information and communication technology on the gender dimension is not analyzed earlier.

**Table 6:** Results of ICT and gender (female labor force).

		Gender equality (female labor force)			Gender equality (female labor force)		
		RE estimation			RLS estimation		
		Model 1	Model 2	Model 3	Model 1	Model 2	Model 3
*Mobile phone use*		0.004 (0.308)	0.024 (0.003)*	0.033 (0.000)*	-0.028 (0.118)	0.028 (0.000)*	0.034 (0.000)*
*Tourism*		-	0.017 (0.080)***	0.212 (0.000)*	-	0.222 (0.000)*	0.202 (0.000)*
*Human capital*		-	-	0.211 (0.000)*	-	-	0.191 (0.000)*
*Trend*		0.001 (0.063)**	0.002 (0.019)**	0.014 (0.000)*	-0.045 (0.003)*	0.029 (0.000)*	0.015 (0.003)*
*Constant*		1.655 (0.000)*	1.787 (0.000)*	0.841 (0.000)*	-	-	-
*R-square*		0.473	0.542	0.628	0.428	0.674	0.602
*Observations*		84	84	84	84	84	84

Notes: The estimated coefficients are shown with probability values in brackets. RE is a Random effect. RLS is Robust Least Square.

* is p<0.01

** is p<0.05 and

*** is p<0.1.

Consistent with economic theory, we find that human capital has a significant positive relationship with gender equality. The human capital theory postulates that education and training are essential determinants of employment and income. An increase in human capital enhances the opportunity to enter the labor force and employment [70, 71]. Therefore, Pacific women must obtain essential training and skills, which would raise their capacity in the job market as well as better their status in the gender dimension. Comparing the results, we find tourism explains most of the change in the female labor force in PICs. Tourism provides better opportunities for women’s workforce participation [19]. For Pacific Island countries tourism is the leading service sector for which these countries not only rely on jobs and foreign exchange but also for long-term economic growth. Based on this result it appears that tourism could play a pivotal role in achieving the objectives of the 2030 agenda for SDG, in particular the commitments to gender equality and empowerment of women [19].

To check for the robustness of our findings, we use female employment instead of the female labor force as the dependent variable to estimate the ICT-gender model. There is a lack of related data on gender equality in Pacific Island countries. There is little in the way of time series data covering the gender dimension of these countries. The results are shown in Table 7 (RE and RLS estimation). Based on the RE estimates, we find mobile phone use separately has a positive but insignificant relationship with female employment. However, in the subsequent regression, which takes into consideration other variables, we established a significant positive relationship. Again, this implies that while ICT alone may not be able to promote gender equality, taken together with other economic fundamentals, ICT could potentially have a significant benefit in the gender dimension. The effect of all other control variables (tourism earning and human capital) are as expected and their coefficients are of similar significance and sign, as reported earlier.

**Table 7:** Results of ICT and gender (female employment).

	Gender equality (female employment)			Gender equality (female employment)		
	RE estimation			RLS estimation		
	Model 1	Model 2	Model 3	Model 1	Model 2	Model 3
*Mobile phone use*	0.002 (0.682)	0.006 (0.003)*	0.012 (0.032)**	0.024 (0.164)	0.028 (0.000)*	0.232 (0.000)*
*Tourism*	-	0.012 (0.028)**	0.243 (0.000)*	-	0.217 (0.000)*	0.197 (0.000)*
*Human capital*	-	-	0.238 (0.000)*		-	0.198 (0.000)*
*Trend*	0.0002 (0.762)	0.0005 (0.541)	0.015 (0.000)*	-0.043 (0.000)*	0.029 (0.000)*	0.018 (0.001)*
*Constant*	1.617 (0.000)*	1.708 (0.000)*	3.735 (0.000)*	-	-	-
*R-square*	0.323	0.445	0.534	0.362	0.644	0.645
*Observations*	84	84	84	84	84	84

Note: The estimated coefficients are shown with probability values in brackets. RE is a Random effect. RLS is Robust Least Square.

* is p<0.01

** is p<0.05 and

*** is p<0.1.

### 5.3. Explaining the potential mechanism of health outcomes of ICT

We now examine model (2) explaining possible mechanisms through which ICT can impact health outcomes in PICs to gain additional insights. The results are reported in Table 8. We use life expectancy as a health indicator as a dependent variable for all the variants of the model (2). In the first column (1) of Table 8, we include the mobile phone variable separately together with other control variables. This is shown mainly for comparison purposes and as found in an earlier analysis that technology–and particularly mobile subscription–is positive and significantly associated with health. The specification in column (2) includes income and gender variables but excludes mobile phone use instead. It is observed that both these variables have a significant and positive relationship with life expectancy. This indicates that income and female empowerment contribute to health outcomes in PICs. We showed earlier that technology use has a positive outcome on gender equality and income [30], suggesting that some of the effects of technology on health pass through the income and gender parity channel. In column (3) of Table 8, we include mobile use together with income and gender variables to examine whether there are other possible channels via which ICT impacts health outcomes. While the estimated coefficient of income almost remains the same, the gender equality variable is insignificant. This is possibly due to controlling for ICT (We do not want to over emphasize the insignificant impact of gender on health, as gender equality does matter health outcome shown in Sekabira and Qaim (2017). We attribute this finding to types of proxies used and estimation techniques in the analysis.). The coefficient of mobile remains consistent and significant, though positive. This is an indication that health outcomes of technology are primarily passing through income and mobile phone use. As discussed above, access and appropriate use of information and communication technology can be a powerful tool for enhancing health and nutrition-related information, which is likely to influence the living habits and health outcomes of the population.

**Table 8:** Possible mechanism through which ICT can impact health.

	Health (life expectancy)					
	RE estimates			RLS estimates		
	(1)	(2)	(3)	(1)	(2)	(3)
*Mobile phone use*	0.002 (0.088)***	-	0.009 (0.000)*	0.016 (0.000)*	-	0.009 (0.000)*
*Income*	-	0.004 (0.001)*	0.019 (0.062)***	-	0.006 (0.000)*	0.004 (0.000)*
*Gender equality*	-	0.010 (0.021)**	0.06 (0.203)	-	0.082 (0.000)*	0.019 (0.050)**
*Health exp*	0.021 (0.000)*	0.033 (0.000)*	0.042 (0.000)*	0.187 (0.000)*	-	0.046 (0.000)*
*Human capital*	0.019 (0.000)*	0.021 (0.000)*	-	0.019 (0.010)**	0.015 (0.000)*	0.018 (0.000)*
*Constant*	-0.171 (0.000)*	0.170 (0.000)*	-0.110 (0.048)**	-	-	-
*R-square*	0.642	0.743	0.621	0.605	0.650	0.684
*Observations*	84	84	84	84	84	84

Notes: The estimated coefficients are shown with probability values in brackets. RE is a Random effect. RLS is Robust Least Square.

* is p<0.01

** is p<0.05 and

*** is p<0.1.

## 6. Conclusion

Information and communication technology have spread quite rapidly in the Pacific region and other emerging and developing worlds. Previous studies have examined the effect of technology on economic indicators such as economic growth, productivity, and prices. However, studies on the technological implication on broader socio-economic development are limited. Understanding socio-economic effects are of great importance, particularly given the need to meet the Sustainable Development Goals set by the United Nations. In this study, we advanced the literature by modeling the impact of ICT on health outcomes and gender aspects in a panel of five Pacific Island Countries from the period 2000 to 2018. Given the data issues with PICs, especially consistent series on socio-economic indicators, we use available data to model this relationship. Health outcome was measured in terms of life expectancy and infant mortality rate. Gender equality was measured in terms of female labor force participation and employment.

Our results, which are robust to a series of tests, show that technology, measured by mobile phone use and internet access is positively associated with life expectancy and reduces the infant mortality rate. The results remain consistent after considering potential confounding economic factors and different estimation methodologies. The impact of mobile phone use on gender equality is also positive, however, this is only after controlling for economic variables such as human capital and tourism. In other words, the effectiveness of technology in gender equality passes through economic fundamentals. This is plausible because technology including mobile phones enables women to get access to information and markets, which they are often constrained with, and with correct economic fundamentals it can be advantageous for women. Further, with respect to a possible mechanism for ICT’s impact on health, while we found that gender equality matters for health, mobile phone use and income are found to be the primary conduit for health outcomes.

The above-mentioned relationships are not only plausible but also constant with economic theory. While the results are robust, we use macroeconomic data that could potentially under-state the measurement issues. The other limitation of the study is the lack of consistent national-level other indicators such as service coverage and health care seeking rate for health outcomes and women empowerment (political, education, health) for gender equality. Once available, future studies can further authenticate the complex co-evolution between health, gender, and technology. Further, future studies using our or other frameworks but with primary data can provide further insights on the role of ICT on health and gender outcomes.

Despite this, we cautiously conclude that technology, particularly mobile phones are a significant determinant of health outcomes and gender equality in Pacific Island Countries. Since the chosen five Pacific Island Countries are typical of the Pacific region and other small economies (in terms of technology adoption, health and gender dimension, and other socio-economic indicators), some policy implications can be drawn. We suggest fostering ICT inclusion in health and gender dimensions of national policy initiatives. Thus, with appropriate ICT infrastructure, education, training, and policy strategies in place, health and gender outcomes can be improved in such economies. Given the relative cost of internet service and mobile phones has declined significantly, the island countries’ connectivity and networking using the various platforms have increased rapidly. This has a positive development impact, which otherwise may not be possible because of the distance, poor physical infrastructure, and low income of societies in the Pacific. Since ICT provides positive spillover effects, its infrastructure cost should be subsidized by the government.
